# CXCL1 derived from tumor-associated macrophages promotes breast cancer metastasis via activating NF-κB/SOX4 signaling

**DOI:** 10.1038/s41419-018-0876-3

**Published:** 2018-08-29

**Authors:** Neng Wang, Weiping Liu, Yifeng Zheng, Shengqi Wang, Bowen Yang, Min Li, Juxian Song, Fengxue Zhang, Xiaotong Zhang, Qi Wang, Zhiyu Wang

**Affiliations:** 1 Integrative Research Laboratory of Breast Cancer, The Research Centre of Integrative Medicine, Discipline of Integrated Chinese and Western Medicine and The Second Affiliated Hospital of Guangzhou University of Chinese Medicine, Guangzhou, Guangdong China; 2 College of Basic Medicine, Guangzhou University of Chinese Medicine, Guangzhou, Guangdong China; 3 Institute of Clinical Pharmacology, Guangzhou University of Chinese Medicine, Guangzhou, China; 4 Post-Doctoral Research Center, Guangzhou University of Chinese Medicine, Guangzhou, Guangdong China; 5 School of Chinese Medicine, Hong Kong Baptist University, Hong Kong SAR, China; 6 Medical College of Acupuncture-Moxibustion and Rehabilitation, Guangzhou University of Chinese Medicine, Guangzhou, China

## Abstract

Tumor-associated macrophages (TAMs) have been implicated in the promotion of breast cancer growth and metastasis, and multiple TAM-secreted cytokines have been identified associating with poor clinical outcomes. However, the therapeutic targets existing in the loop between TAMs and cancer cells are still required for further investigation. Here in, cytokine array validated that C-X-C motif chemokine ligand 1 (CXCL1) is the most abundant chemokine secreted by TAMs, and CXCL1 can promote breast cancer migration and invasion ability, as well as epithelial–mesenchymal transition in both mouse and human breast cancer cells. QPCR screening further validated SOX4 as the highest responsive gene following CXCL1 administration. Mechanistic study revealed that CXCL1 binds to SOX4 promoter and activates its transcription via NF-κB pathway. In vivo breast cancer xenografts demonstrated that CXCL1 silencing in TAMs results in a significant reduction in breast cancer growth and metastatic burden. Bioinformatic analysis and clinical investigation finally suggested that high CXCL1 expression is significantly correlated with breast cancer lymph node metastasis, poor overall survival and basal-like subtype. Taken together, our results indicated that TAMs/CXCL1 promotes breast cancer metastasis via NF-κB/SOX4 activation, and CXCL1-based therapy might become a novel strategy for breast cancer metastasis prevention.

## Introduction

Breast cancer is the most frequent cancer among women and the second most common cause of cancer deaths worldwide, with an estimated 1.67 million new cases and 521,900 premature deaths in 2012^1^. Although advances have been made in novel drug discovery and therapeutic strategies, breast cancer death events will approach 560,407 in 2020^1^. Distant metastasis is responsible for ~ 90% of breast cancer-related deaths^2^, so identifying metastatic targets is of great interest. Recent evidence has suggested that metastasis involves a network of interactions between numerous cellular components and cytokines^3^. Because their interaction and crosstalk might lead to the formation of a tumor microenvironment (TME) that contributes to tumor progression, so it is important to identify the key molecular events by which stromal cells regulate cancer metastasis.

Macrophages are the most abundant stromal cells associated with the host immune system in multiple malignancies. They are reportedly involved in cancer onset, and progression, and exist as classically activated macrophages (M1) and alternatively activated macrophages (M2 or tumor-associated macrophages, TAMs)^4^. M1 macrophages are activated when exposed to lipopolysaccharides, interferon-γ and tumor necrosis factor-alpha. However, when treated with interleukin (IL-4) and IL-13, they are polarized to an immunosuppressive M2 phenotype and are involved in cancer progression. Recently, the density of TAMs was found to correlate with a poor prognosis in multiple malignancies, including breast cancer^5^. For example, an increase in the percentage of M2 macrophages was associated with poor patient survival in esophageal adenocarcinoma^6^. TAM intensity is also involved in resistance to androgen blockade therapy in prostate cancer^7^. Moreover, the macrophage-stimulating protein pathway promotes breast cancer metastasis and predicts a poor prognosis^8^. Pharmacological macrophage inhibition by clodronate was found to decrease lung metastasis in pancreatic cancer xenografts^9^, and M2 macrophage polarization was confirmed to be critical for the chemopreventive effects of various phytochemicals, such as curcumin, fenretinide and resveratrol^9–11^. Thus, targeting stromal TAMs may be promising for preventing cancer development and metastasis.

Chemokines are critical secretors derived from TAMs that mediate cancer progress and metastasis. To date, approximately 50 chemokines have been identified, and several TAM-derived chemokines are associated with tumor progression. In prostate cancer, TAMs promote cancer migration through the release of CC chemokine ligand 22 (CCL22)^12^. C-X-C motif chemokine ligand 8 (CXCL-8) is secreted by TAMs and is correlated with the increased metastatic potential of thyroid papillary cancer^13^. It has also been shown that breast cancer metastasis can be mediated by CCL18 secretion in TAMs by activating PITPNM3^14^. With regard to CXCL1, several studies have highlighted its significant role in mediating the communication between cancer cells and TME. Breast cancer cell-secreted CXCL1 recruits CD11b^+^Gr1^+^ myeloid cells into the tumor, thereby supporting cancer survival and metastasis by activating calprotectin expression^15^. Meanwhile, CXCL1–CXCR2 axis is overactivated in gastric cancer and is closely correlated with the migration and invasion ability of malignant cells^16^. By comparing the profiles of secreted proteins in low- and high-grade invasive balder cancer, CXCL1 was identified as the most significantly differentially expressed chemokine, with urinary levels that were significantly higher in patients with invasive bladder cancer^17^. Interestingly, CXCL1 levels in bladder cancer tissue were positively associated with TAM infiltration, and CXCL1-expressing TAMs enhanced bladder cancer growth when injected together in nude mice^17^. Thus, CXCL1 signaling in the TME plays a critical role in cancer development and prognosis. However, the abundance of CXCL1 among TAM-secreted chemokines and its level relative to cancer cells remain largely unknown. In addition, the molecular mechanisms underlying the promotion of cancer metastasis by CXCL1 are also unknown. Therefore, additional research studies are urgently required on the CXCL1-mediated crosstalk between TAMs and cancer cells.

The current study was designed to investigate the level and molecular mechanisms of TAM-derived CXCL1 in promoting breast cancer metastasis. By chemokine profiling, we validated CXCL1 as the most abundant secretor released from TAMs, and CXCL1 administration promoted breast cancer metastasis via the nuclear factor kappa-light-chain-enhancer of activated B cells (NF-κB)/sex determining region Y-box 4 (SOX4) signaling in both murine and human models. CXCL1 silencing in TAMs significantly inhibited breast cancer growth and metastasis. High CXCL1 expression was associated with advanced cancer stage, lymph node (LN) metastasis and poor survival. These data not only reveal the underlying mechanisms by which CXCL1 mediates crosstalk between TAMs and breast cancer cells but also suggest that CXCL1 may be a potential therapeutic biomarker in TAM for the prevention of metastasis.

## Results

### TAM-derived CXCL1 is overexpressed in lung metastatic lesions of breast cancer

**Fig. 1:**
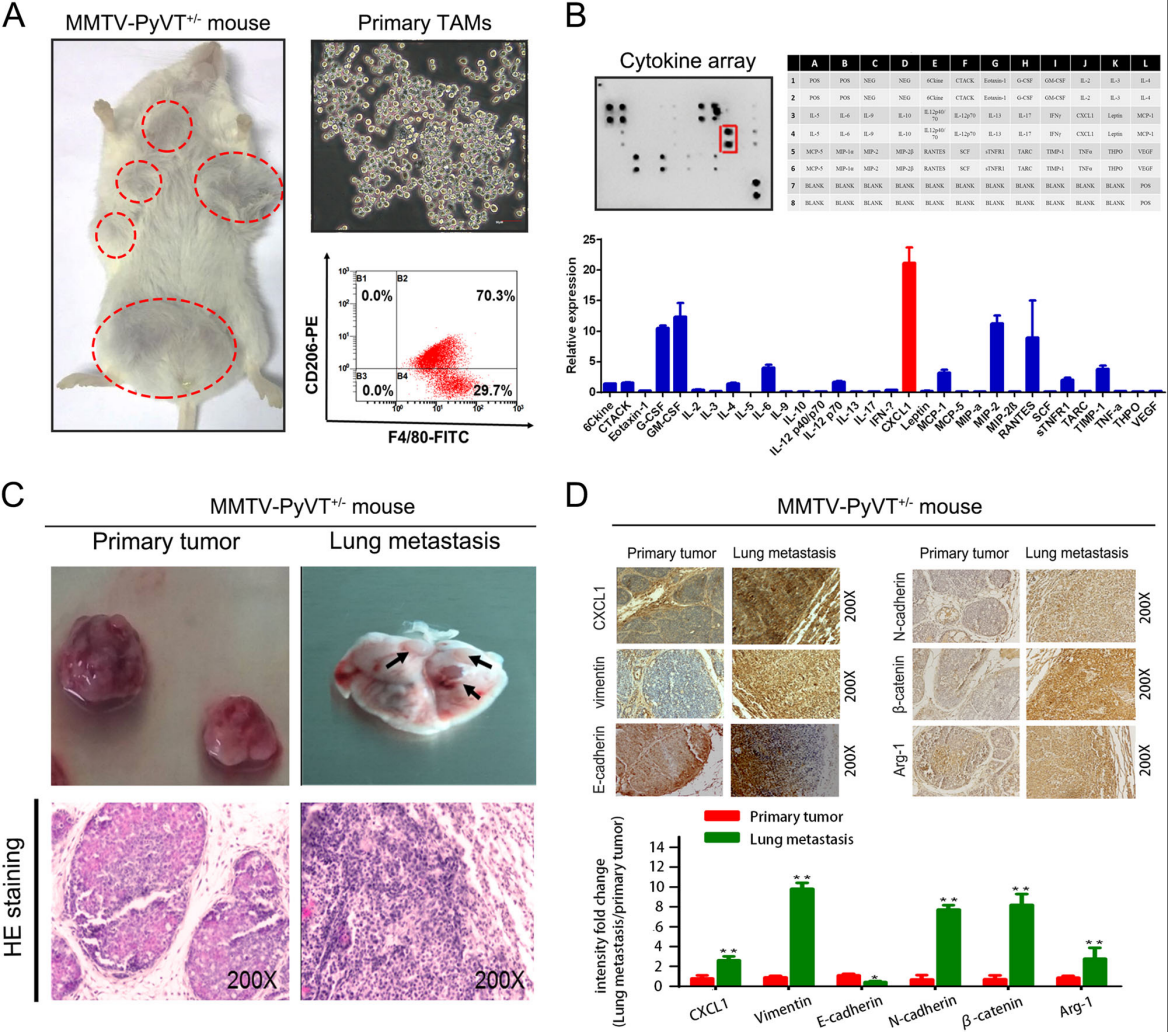
TAM-secreted CXCL1 is highly increased in the lung metastatic lesion of breast cancer. **a** TAMs were isolated from breast tumors by differential adhesion technique and validated by F4/80^+^/CD206^+^ staining. **b** Cytokine array revealed that CXCL1 had the highest expression in the supernatants of TAMs. **c** Primary mammary tumors and lung metastatic lesions were collected from MMTV-PyVT^+/-^ mice, respectively, and validated by HE staining. **d** CXCL1 expression was significantly enhanced in the lung metastatic lesions compared with its primary tumors, accompanied by increased expression levels of vimentin, N-cadherin and β-catenin, whereas the epithelial marker E-cadherin was reduced, indicating that CXCL1 expression was closely correlated with EMT process. Meanwhile, ARG1 expression was also increased in the lung metastasis lesions, implying that the enhanced CXCL1 expression might be correlated with increased M2 macrophage phenotype. (All values from three independent experiments are quantified as mean ± SD, *P < 0.05, **P < 0.01)

TAMs from the breast tumors of MMTV-PyMT^+/-^ mice were isolated using the differential adhesion technique described in the Materials and methods section. Flow cytometry demonstrated that ~ 70% isolated cells comprised the F4/80^+^CD206^+^ population; the morphology of the TAMs is shown in Fig. 1a. Figure 1b shows that among the 32 cytokines, CXCL1 was the most abundant. To determine the role of CXCL1 in mediating breast cancer metastasis, the primary mammary tumor and its lung metastatic lesions were collected. CXCL1 was significantly elevated in the metastatic lesions, accompanied by the increased expression of arginase 1 (Arg-1) and epithelial–mesenchymal transition (EMT)-related markers including β-catenin, vimentin and N-cadherin. Meanwhile, the decreased expression of the epithelial marker E-cadherin in lung metastatic tissue was also observed (Figs. 1c, d). Thus, CXCL1 might be a mediator of breast cancer metastasis.

### CXCL1 promotes mouse breast cancer cell metastatic ability

**Fig. 2:**
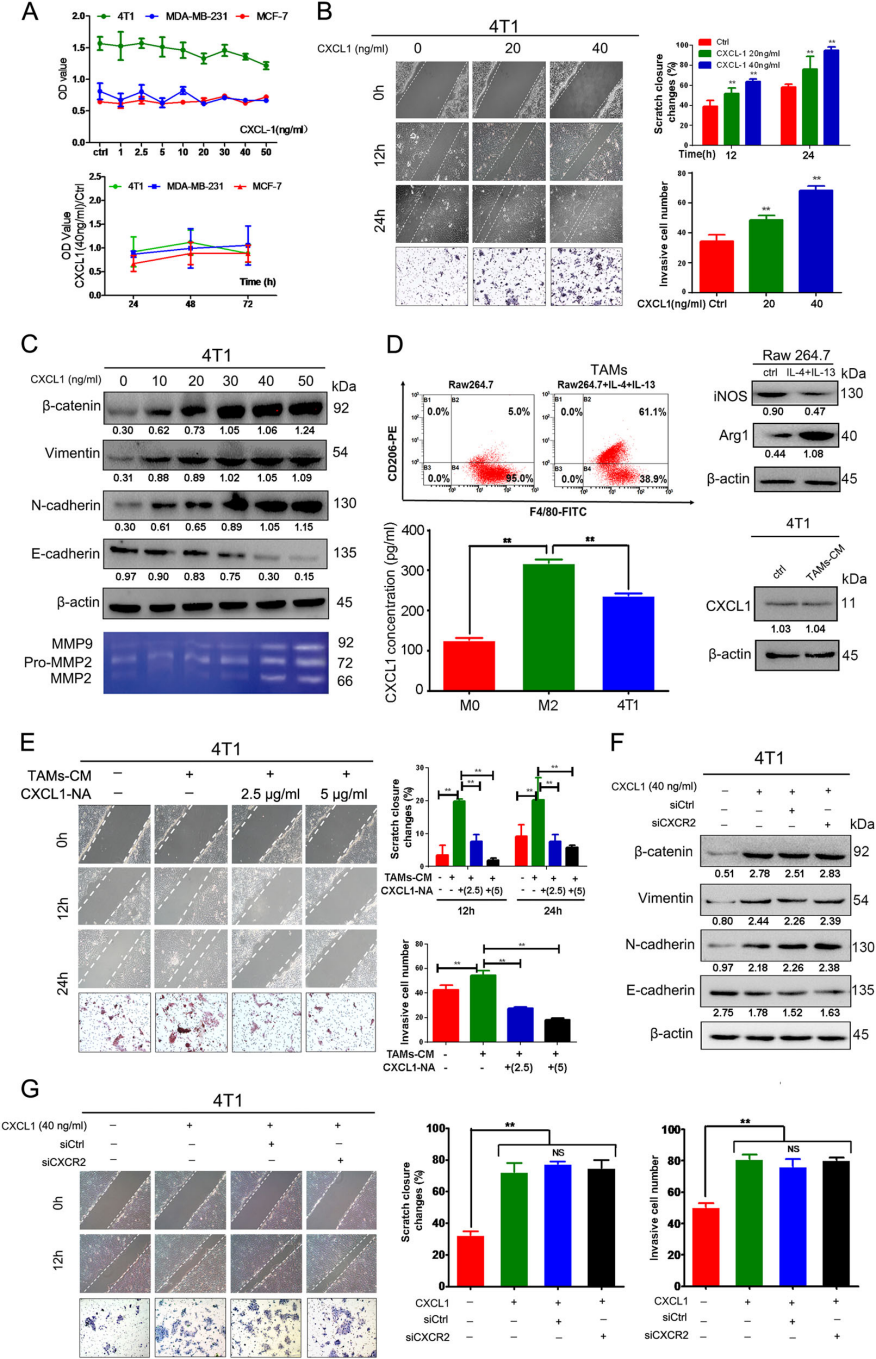
TAM-secreted CXCL1 promotes mouse breast cancer cells migration and invasion. **a** CXCL1 had little influence on the proliferation of breast cancer cells including 4T1, MDA-MB-231 and MCF-7 in both dose- and time-dependent manner. **b** Would-healing and Transwell assay revealed that CXCL1 could promote the migration and invasion ability of 4T1 cells. **c** Western blotting results showed that CXCL1 administration promotes the EMT process of 4T1 cells, presenting as dose-dependent increase of vimentin, N-cadherin, β-catenin and gradual downregulation of E-cadherin. Gelatin zymography also indicated that CXCL1 treatment results in increased secretion of MMP-9 and MMP-2 from 4T1 cells. **d** The mouse macrophage cell line Raw264.7 was induced to TAMs by administrating IL-4 and IL-13, resulting in the increased expression of CD206 and Arg-1, and reduction of iNOS; Meanwhile, ELISA assay demonstrated that CXCL1 expression in M2-Raw264.7 supernatants was significantly higher than that in either M0-Raw164.7 or 4T1 cancer cells. Notably, the CM of TAMs did not increase CXCL1 expression in 4T1 cancer cells. **e** TAMs-CM treatment led to increased migration and invasion ability of 4T1 cells, whereas was blocked by administration of CXCL1-neutralizing antibody. **f,** **g** CXCR2 silencing in 4T1 cancer cells did not block CXCL1-induced EMT and invasion ability. (All values from three independent experiments are quantified as mean ± SD, **P < 0.01)

To determine whether exogenous CXCL1 could promote breast cancer metastasis, we added the CXCL1 cytokine to cells, and found that 0–50 ng/mL CXCL1 had little influence on the cell proliferation of the 4T1, MDA-MB-231 and MCF-7 breast cancer cells (Fig. 2a). Wound-healing and Transwell assays showed that CXCL1 significantly increased 4T1 cell migration and invasiveness (Fig. 2b). Western blot analysis showed that after CXCL1 treatment, β-catenin, vimentin and N-cadherin expression significantly increased but E-cadherin expression was gradually inhibited, indicating that the EMT process was activated by CXCL1 (Fig. 2c). A gelatin zymography assay demonstrated that metalloproteinase 2 (MMP-2) and MMP-9 secreted by 4T1 cells were increased with CXCL1 administration, validating the pro-metastatic ability of CXCL1 (Fig. 2c). Next, we induced the M2 phenotype transition of Raw264.7 macrophages with IL-4 and IL-13 to increase expression of the M2 biomarkers cluster of differentiation 206 (CD206) and Arg-1 and inhibit inducible nitric oxidase synthase (Fig. 2d). Meanwhile, it was demonstrated that CXCL1 level in the supernatants of M2-Raw264.7 macrophages was significantly higher than that in M1 phenotype or 4T1 cancer cells, and the conditional medium (CM) collected from M2-Raw264.7 did not result in the increased expression of CXCL1 in 4T1 cancer cells, indicating that CXCL1 derived from TAMs may be an independent factor that influences breast cancer metastasis (Fig. 2d). Notably, the CM of M2-Raw264.7 macrophages were found to promote the cell migration and invasiveness of 4T1 cells, as determined by wound-healing and Transwell assays. However, when CXCL1-neutralizing antibody was added to the co-culture system, CM-induced invasion was blocked in a dose-dependent manner, indicating that CXCL1 might be critical to TAM-induced aggressiveness (Fig. 2e). To determine whether CXCL-induced aggressiveness was dependent on CXCR2 expression in breast cancer cells, CXCR2 was knocked down in 4T1 cells (Supplementary Fig. 1). Western blotting revealed that CXCL1-induced EMT was not blocked following CXCR2 silencing (Fig. 2f). Similarly, wound-healing and Transwell results also showed that CXCR2 silencing had little effect on CXCL1-induced aggressiveness (Fig. 2g), indicating that CXCL1-activated invasiveness was CXCR2 independent.

### CXCL1 enhances the invasiveness and EMT in human breast cancer cells

**Fig. 3:**
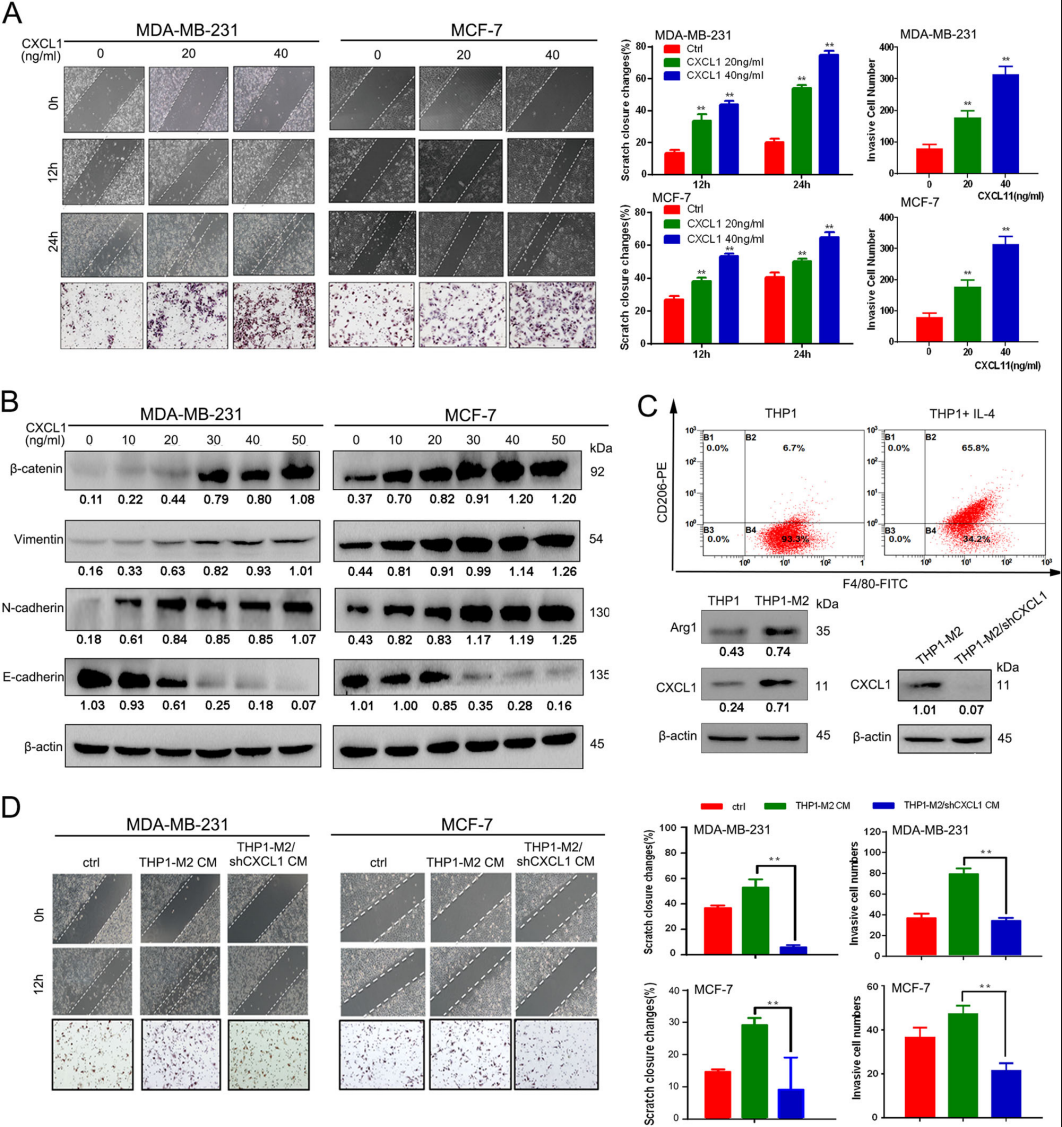
TAM-secreted CXCL1 promotes human breast cancer cells migration and invasion. **a** Would-healing and Transwell assay revealed that CXCL1 could promote the migration and invasion ability of both MDA-MB-231 and MCF-7 cells. **b** Western blotting results showed that CXCL1 administration dose dependently promotes the EMT process of both breast cancer cells, presenting as increased expression of vimentin, N-cadherin, β-catenin and gradual downregulation of E-cadherin. **c** The enhanced expression levels of CD206, Arg1 and CXCL1 validated the successful induction of TAMs by administrating IL-4. Meanwhile, shCXCL1 was applied to knockdown its expression in M2-THP1 macrophages. **d** CXCL1 silencing in M2-THP1 macrophages suppressed the enhanced migration and invasion abilities of MDA-MB-231 and MCF-7 cells induced by TAMs-CM. (All values from three independent experiments are quantified as mean ± SD, **P *<* 0.01)

We used MDA-MB-231 and MCF-7 cells to determine if CXCL1 had similar effects on human breast cancer and found that CXCL1 promoted cancer cell migration and invasiveness (Fig. 3a). Western blot analysis validated that CXCL1 induced the EMT in both cell lines, concomitantly with a decrease in E-cadherin expression and an increase in β-catenin, vimentin and N-cadherin expression (Fig. 3b). Then, we induced the M2 polarization of human THP1 macrophages by adding IL-4, and flow cytometry and western blotting demonstrated that the expression of the M2 phenotype markers CD206 and Arg1 was significantly increased. Similar to our findings in mouse TAMs, CXCL1 expression was also upregulated in M2-THP1 macrophages. To examine the role of TAM-derived CXCL1 in mediating breast cancer aggressiveness, CXCL1 expression in M2 phenotype THP1 macrophages was knocked down by transfection of its short hairpin RNA (shRNA) plasmid (Fig. 3c). Wound-healing and Transwell assays showed that CXCL silencing inhibited the invasion ability of cancer cells induced by the CM from THP1 cells (Fig. 3d). These findings validated the in vitro invasive-promoting effects of TAM-secreted CXCL1 in mouse and human breast cancer models.

### Validation of SOX4 as a downstream response gene after CXCL1 treatment

**Fig. 4:**
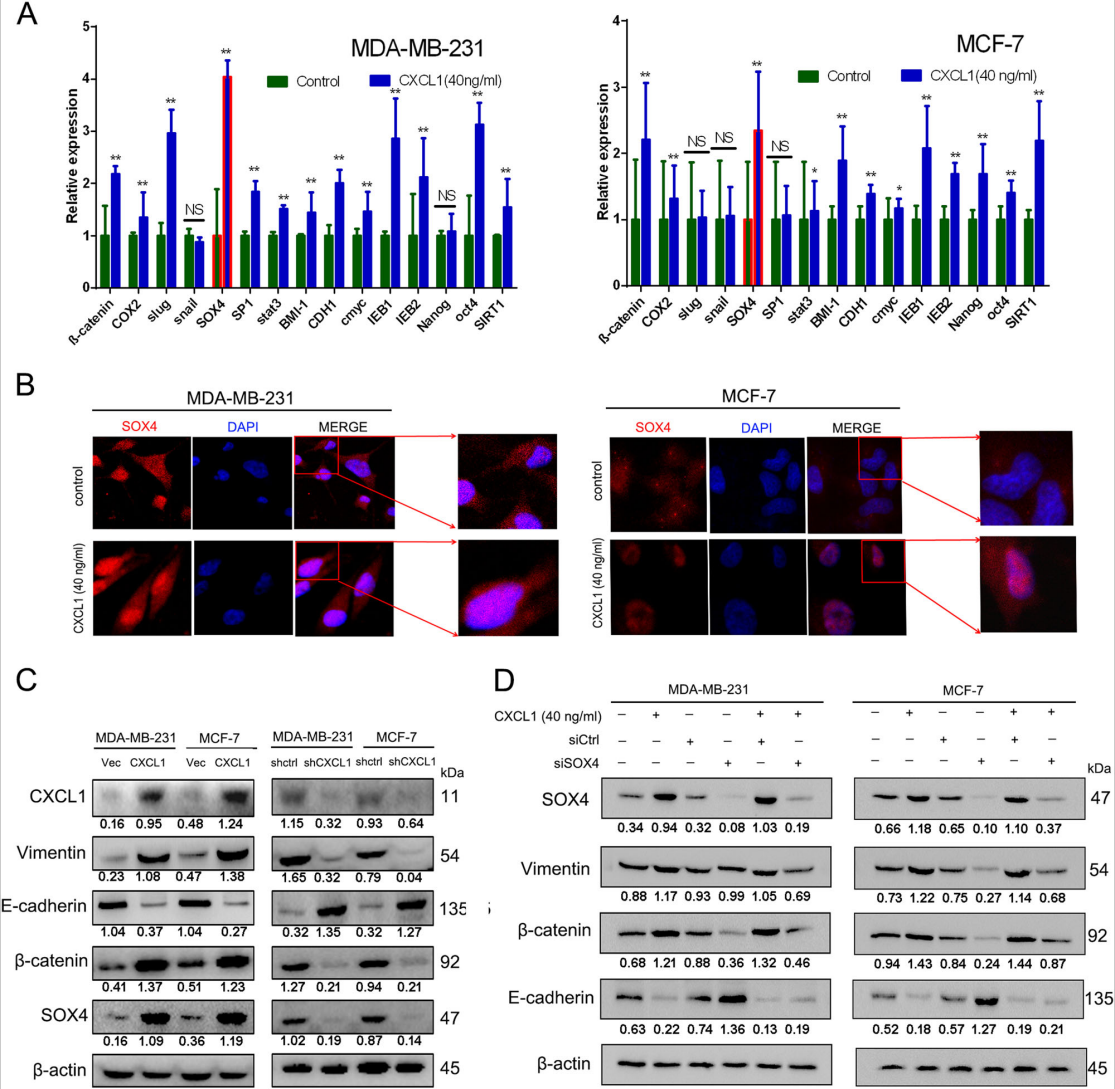
CXCL1 promotes breast cancer EMT process via activating SOX4 signaling. **a** qPCR screening assay revealed SOX4 as the highest responsive gene following CXCL1 administration in both MDA-MB-231 and MCF-7 cells. **b** Immunofluorescence assay revealed that CXCL1 administration resulted in increased SOX4 expression and its nucleus transportation ( × 400). **c** Western blotting assay indicated that CXCL1 overexpression resulted in SOX4 upregulation, accompanied by enhanced EMT process. By contrast, CXCL1 silencing in both breast cancer cells led to the downregulation of SOX4 and inhibited EMT process. **d** SOX4 silencing inhibited the EMT process in both breast cancer cells induced by CXCL1 administration, presenting as decreased expression of vimentin, β-catenin and upregulation of E-cadherin. (All values from three independent experiments are quantified as mean ± SD, **P *<* 0.01 vs. control)

Because cancer stem cells (CSCs) are reportedly the root of cancer recurrence and metastasis, we determined if CXCL1 could increase the population of CSCs in breast cancer cells. Flow cytometry data showed that CXCL1 treatment did not increase CD44^+^/CD24^-^ or aldehyde dehydrogenase assays (ALDH^+^) in the MDA-MB-231 and MCF-7 breast cancer cell lines (Supplementary Figure 2), indicating that CSC-enhancing effects may not explain CXCL1-induced metastasis. Subsequently, we screened the expression of a panel of metastasis-related genes after CXCL1 treatment using quantitative PCR (primer sequences are listed in Supplementary Table 1), and found that 11 metastatic genes were significantly elevated (Fig. 4a), of which SOX4 was the most elevated. We also used immunofluorescence to determine the effects of CXCL1 on SOX4 signaling, and found that after CXCL1 treatment, SOX4 expression significantly increased in the nucleus of both breast cancer cell lines (Fig. 4b). Thus, SOX4 might function as the most significant responsive gene following CXCL1 administration.

### CXCL1-mediated EMT is NF-κB/SOX4 dependent

**Fig. 5:**
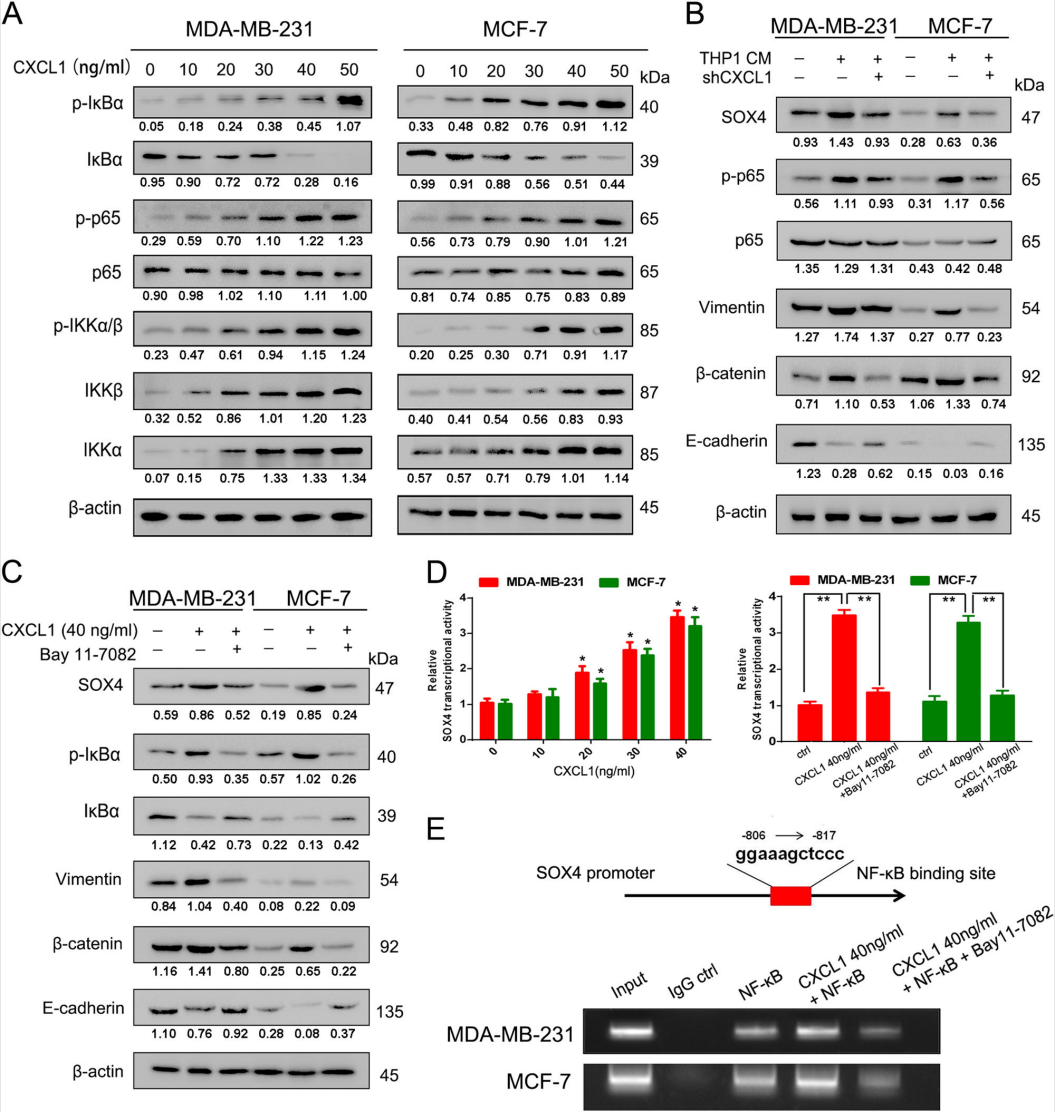
CXCL1 activates SOX4 transcription via NF-κB signaling. **a** CXCL1 administration resulted in the activated NF-κB signaling in both breast cancer cells, presenting as increased expression levels of p-IKBα, p-P65/P65, p-IKKα/β, IKKα and IKKβ. **b** CXCL1 silencing in breast cancer cells suppressed p-P65/P65 activation, accompanied by decreased expression of SOX4 and EMT blockade. **c** NF-κB inhibitor Bay 11–7082 treatment inhibited the activation of SOX4 and EMT process induced by CXCL1. **d** CXCL1 dose dependently increased SOX4 transcription, whereas Bay 11–7082 treatment significantly inhibited the process. **e** CHIP assay demonstrated that NF-κB could bind with the SOX4 promoter region to activate its transcription, which was enahnced by CXCL1 administration but reversed by Bay 11–7082 treatment. (All values from three independent experiments are quantified as mean ± SD, *P < 0.05, **P *<* 0.01)

To evaluate the regulatory relationship between CXCL1 and SOX4, we upregulated or inhibited CXCL1 expression in MDA-MB-231 and MCF-7 cells by genetic interference. When CXCL1 was overexpressed in breast cancer cells, SOX4 expression was concomitantly increased. Conversely, when CXCL1 was silenced, SOX4 expression was downregulated correspondingly (Fig. 4c). To confirm the critical role of SOX4 in mediating CXCL1-induced invasion ability, SOX4 expression in both breast cancer cells was silenced by transfecting its small interfering RNA (siRNA) (Supplementary Fig. 3). Western blotting showed that CXCL1 treatment increased SOX4 expression and facilitated the EMT. However, SOX4 silencing reduced vimentin and β-catenin expression and increased E-cadherin. In addition, SOX4 silencing blocked the EMT-promoting effects induced by CXCL1 in MDA-MB-231 and MCF-7 cells, indicating that CXCL1 might induce the EMT via SOX4 activation (Fig. 4d). Because NF-κB was reported to be a transcription factor of SOX4^18^, after treating cells with CXCL1, we examined the NF-κB pathway activity. Western blotting showed that with increasing concentrations of CXCL1, the expression of IKKα (IκB kinase α) and IKKβ and their phosphorylated form p-IKKα/β was upregulated in breast cancer cells, which induced dissociation of IκBα from p65 and led to p65 phosphorylation (Fig. 5a). Thus, CXCL1 acts as an upstream factor of the NF-κB pathway. Meanwhile, CXCL1 silencing in THP-1 macrophages reduced SOX4 and phosphorylated p65 (p-p65), which was accompanied by the decreased expression of β-catenin and vimentin but the increased E-cadherin. These data suggest that CXCL1 is a critical cytokine in THP1 CM-mediated activation of the NF-κB/SOX4 pathway (Fig. 5b). To validate the significance of the NF-κB pathway in mediating CXCL1-induced SOX4 activation, the IκBα inhibitor Bay 11–7082 was added to the cell culture system. The results showed that CXCL1-induced SOX4 overexpression was blocked by Bay 11–7082 treatment, accompanied by the increased expression of E-cadherin and reduction of vimentin and β-catenin (Fig. 5c), suggesting that the NF-κB pathway is critical in mediating CXCL1-induced metastasis. The quantitative PCR (qPCR) results also confirmed that CXCL1 could activate SOX4 transcription in a dose-dependent manner, whereas Bay 11–7082 administration inhibited CXCL1-induced SOX4 mRNA expression, indicating that CXCL1 might activate SOX4 transcription via NF-κB signaling (Fig. 5d). The chromatin immunoprecipitation assay was conducted to validate that NF-κB could interact with the predicted binding region on the SOX4 promoter. The results showed that NF-κB could bind to the promoter region of SOX4, and that CXCL1 administration directly enhanced NF-κB-binding activity, whereas Bay 11–7082 treatment significantly inhibited the CXCL1-induced NF-κB/SOX4 interaction (Fig. 5d). Taken together, CXCL1 activates SOX4 transcription and subsequent EMT via the NF-κB pathway.

### CXCL1 knockdown in TAMs inhibits breast cancer growth and lung colonization

**Fig. 6:**
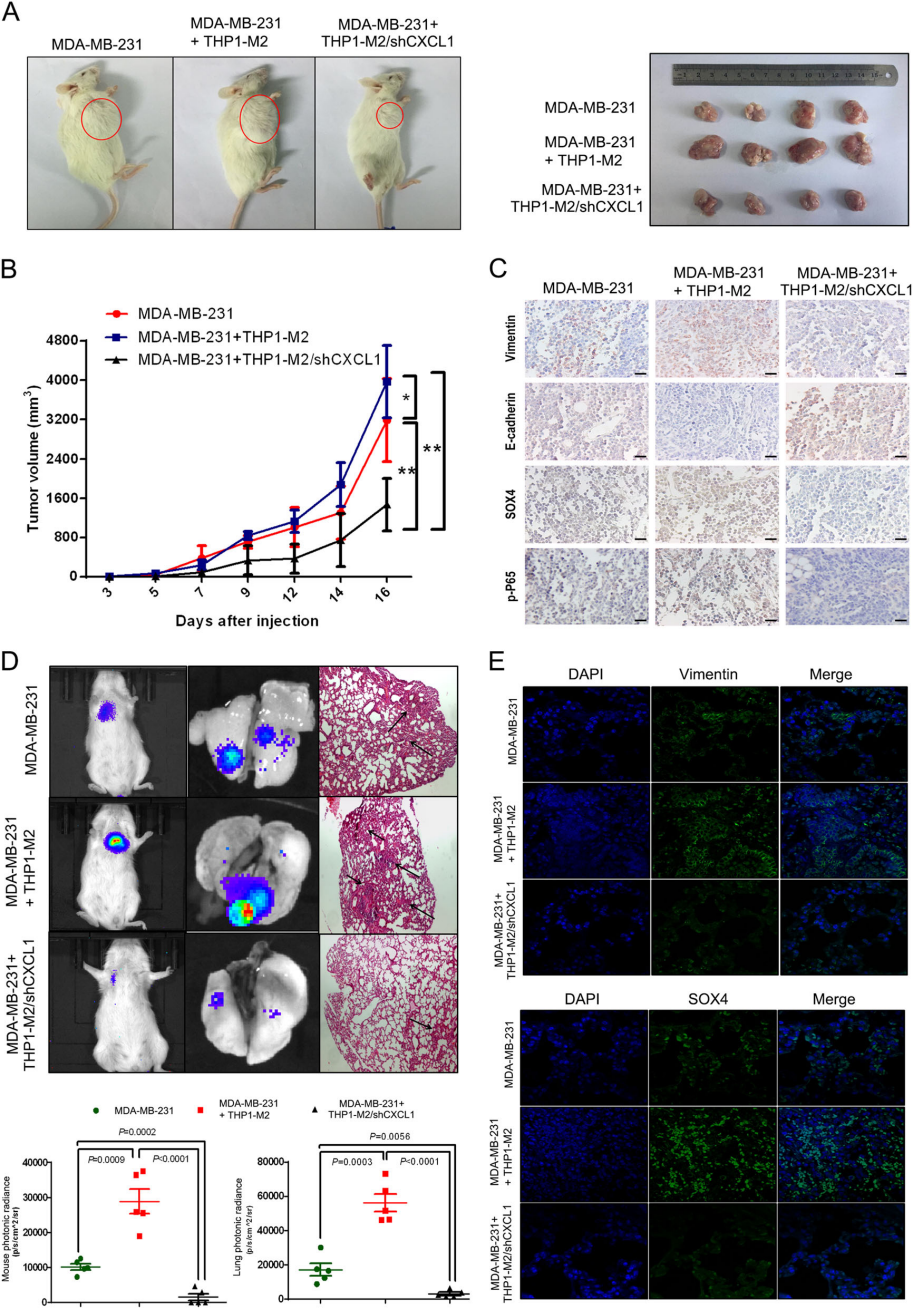
CXCL1 silencing inhibits TAM-induced breast cancer growth and lung metastasis. **a**, **b** In situ breast cancer xenograft showed that TAMs co-injection promotes breast cancer growth, whereas CXCL1 silencing in TAMs significantly suppressed tumor growth (values represents as mean ± SD, *n* = 4, *P < 0.05, **P < 0.01). **c** Immunohistochemistry assay showed that TAMs co-injection significantly elevates the expression of vimentin, SOX4 and p-P65 in tumors, whereas E-cadherin expression was inhibited. By contrast, CXCL1 silencing in TAMs led to opposite effects (scale bars indicate 50 μm). **d** In vivo luciferase imaging model showed that TAMs significantly promotes the metastatic colonies formation, whereas CXCL1 silencing in TAMs significantly blocks breast cancer lung colony growth. **e** Immunofluorescence assay revealed that TAMs co-injection significantly elevates vimentin and SOX4 expression in the metastatic lesions, whereas CXCL1 silencing in TAMs results in an inhibition of vimentin and SOX4 expression ( × 400)

Using an orthotropic breast cancer xenograft model in NOD/SCID mice, IL-4-treated THP-1 or THP-1/shCXCL1 cells were co-injected with MDA-MB-231 cancer cells into the mammary fat pads of NOD/SCID mice at a ratio of 1:3. We found that THP-1 significantly promoted breast cancer growth. However, CXCL1 knockdown in THP-1 cells not only blocked the growth-promoting effects of macrophages, but also significantly reduced breast cancer growth compared with control mice (Figs. 6a, b). Because CXCL1 knockdown in THP-1 cells did not influence macrophages proliferation and invasion (Supplementary Fig. 4), tumor regression might have been due to subsequent reactions of cancer cells responsive to CXCL1 knockdown in macrophages. Immunohistochemistry results revealed that CXCL1 knockdown increased the expression of E-cadherin but reduced vimentin, SOX4 and p-p65 expression, consistent with our in vitro findings (Fig. 6c and Supplementary Fig. 5). To investigate whether CXCL1 silencing in TAMs would inhibit cancer cell colonization in distant lung tissue, we used the experimental tail vein injection method to create a colonization model. After 2 months, in vivo bioluminescent imaging and and hematoxylin and eosin staining confirmed that microcolonization lesions were more extensive in the THP1 co-injection group, but were significantly suppressed after CXCL1 knockdown (Fig. 6d). The immunofluorescence results also validated that CXCL1 silencing suppressed the expression levels of SOX4 and vimentin in the lung lesions (Fig. 6e). Thus, TAMs/CXCL1 may be promising therapeutic targets for inhibiting breast cancer metastasis to the lungs.

### CXCL1 expression is correlated with the clinicopathological characteristics and prognosis of breast cancer

**Fig. 7:**
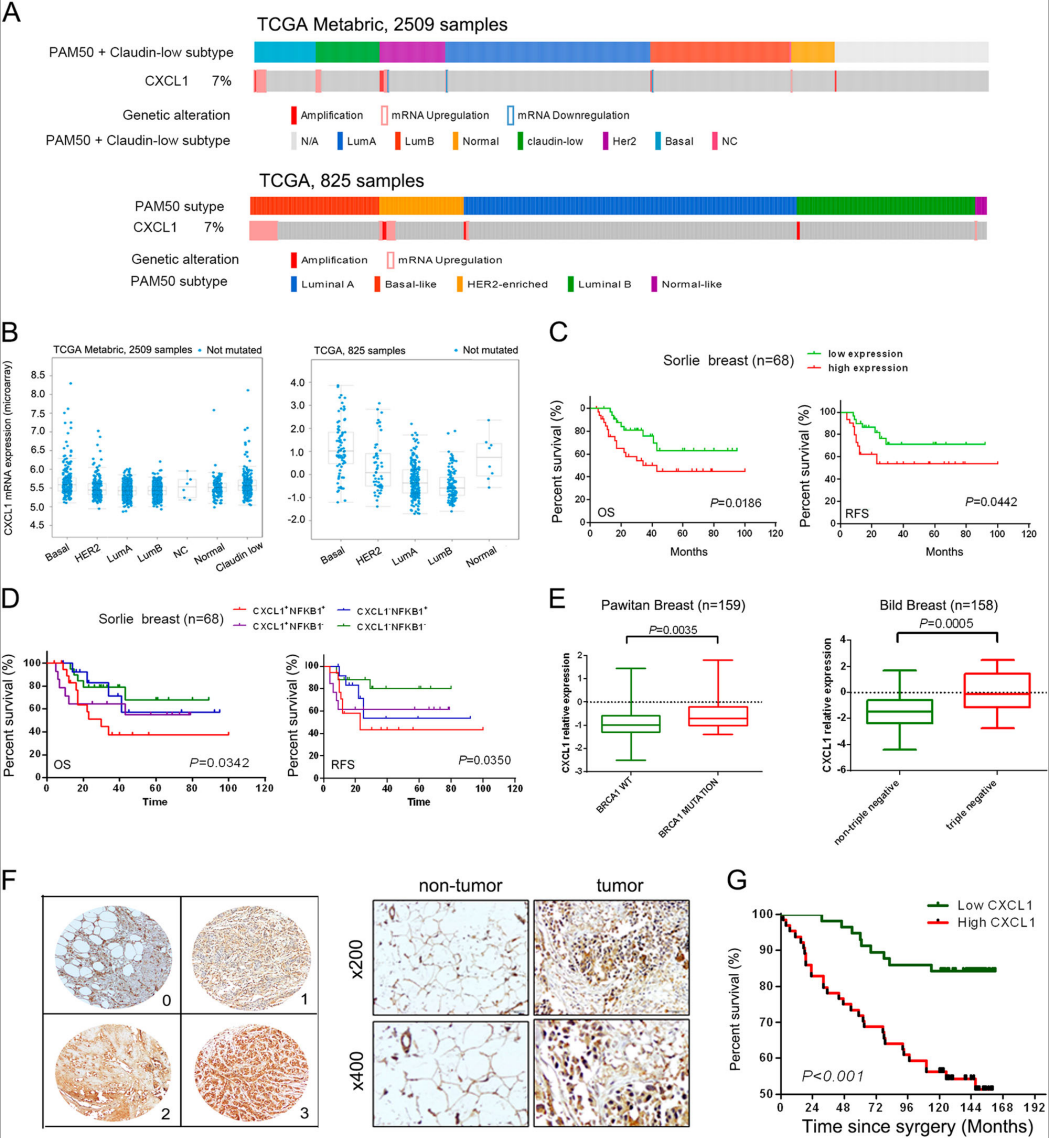
Clinical significance of CXCL1 in predicting breast cancer prognosis. **a** The OncoPrint tab summarizes the genomic alterations of CXCL1 across the sample set TCGA and Metabric. CXCL1 had 4 and 7% genetic alterations in Metabric and TCGA studies, respectively. **b** The expression of CXCL1 mRNA in each type of breast cancer among Metabric and TCGA corhot, respectively. The results showed that CXCL1 had relatively higher expression in basal-like breast cancer in both studies. **c** Sorlie breast study in ONCOMINE database showed that CXCL1 high expression was correlated with poor OS (*P* = 0.0186) and RFS (*P* = 0.0442) of breast cancer. **d** Breast cancer patients with CXCL1^+^/NF-κB^+^ had the poorest OS (*P* = 0.0342) and RFS (*P* = 0.0350) in Sorlie study. **e** Pawitan breast study suggested that CXCL1 expression in BRCA1 mutated populations had significant increase (*P* = 0.0035), and Bild breast study indicated that triple-negative breast cancer patients had higher CXCL1 expression (*P* = 0.0005). **f, g** Tissue microarray analysis showed that CXCL1 had higher expression in tumor tissues compared with normal mamamry tissues ( × 400), and CXCL1 high expression was also correlated with a poor OS (*P* < 0.001)

To confirm the clinical significance of CXCL1, we measured the expression of CXCL1 in The Cancer Genome Atlas database from ~ 3000 patients. Figure 7a shows that CXCL1 amplification or overexpression occurred in 4–7% of breast cancer patients; most with upregulated CXCL1 expression were classified as basal like. Compared with other breast cancer subtypes, basal-like subtypes had the highest mean CXCL1 expression according to the TCGA databases (Fig. 7b). Using the Oncomine database, the Sorlie breast study suggested that high CXCL1 expression was significantly correlated with overall survival (OS, *P* = 0.0186) and recurrence-free survival (RFS, *P* = 0.0442)^19^, indicating that high CXCL1 expression might predict a poor prognosis of breast cancer (Fig. 7c). Meanwhile, breast cancer patients with both high CXCL1 and NF-κB expression had the poorest OS and RFS (*P* = 0.0342 and *P* = 0.0350, respectively, Fig. 7d). However, combinational analysis CXCL1 and SOX4 did not result in clinical significance in OS and RFS analysis (Supplementary Fig. 6). Breast cancer studies by Pawitan and Bild demonstrated that CXCL1 expression had a significant increase in the BRCA1 mutant (*P* = 0.0035) and triple-negative (*P* = 0.0005) breast cancer patients, respectively^20,21^ (Fig. 7e). A breast cancer tissue microarray further validated that CXCL1 was more expressed in breast cancer tissue compared with normal adjacent tissue (Fig. 7f and Supplementary Fig.7). In the breast cancer tissue from 121 patients, we examined whether CXCL1 expression was associated with clinicopathological parameters. The mean CXCL1 staining scores indicated that 64 patients had high CXCL1 and 57 patients had low CXCL1 expression, and Table 1 shows that the expression of CXCL1 was positively associated with LN status (*P* = 0.001), TNM stage (*P* < 0.001) and LN infiltration (*P* = 0.016). No significant correlation existed between CXCL1 expression and other clinicopathological factors (Table 1). Multivariate Cox regression analysis showed that patients with high CXCL1 expression had poor OS (Fig. 7g and Table 2). Thus, CXCL1 is closely correlated with breast cancer metastasis and survival.

**Table 1:** Relationships between CXCL1 and clinicopathologic characteristics

Characteristic	Cases	CXCL1 expression				*p*-Value
		High	No. (%)	Low	No. (%)	
Age (years)						0.618
<50	56	31	55.35714	25	44.64286	
≥50	65	33	50.76923	32	49.23077	
Histologic grade						0.42
G1	11	6	54.54545	5	45.45455	
G2	101	53	52.47525	48	47.52475	
G3	6	5	83.33333	1	16.66667	
Tumor size (T)						0.471
T1	24	13	54.16667	11	45.83333	
T2	85	42	49.41176	43	50.58824	
T3	12	9	75	3	25	
Lymph node status (*N*)						0.001*
N0	40	15	37.5	25	62.5	
N1	40	19	47.5	21	52.5	
N2	31	23	74.19355	8	25.80645	
N3	9	7	77.77778	2	22.22222	
TNM stage						< 0.001*
I	7	2	28.57143	5	71.42857	
II	70	30	42.85714	40	57.14286	
III	43	32	74.4186	11	25.5814	
LN infiltration						0.016*
Yes	79	48	60.75949	31	39.24051	
No	40	15	37.5	25	62.5	
ER						0.27
Positive	73	35	47.94521	38	52.05479	
Negative	46	23	50	16	34.78261	
PR						0.38
Positive	67	33	49.25373	34	50.74627	
Negative	45	26	57.77778	19	42.22222	
HER2						0.76
Positive	34	17	50	17	50	
Negative	79	42	53.16456	37	46.83544	

^*^p < 0.05, statistically significant

**Table 2:** Prognostic value of CXCL1 for overall survival (OS) by univariate and multivariate analyses

OS	Univariate analysis	Multivariate analysis			
	*p*-Value	HR	95% IC		*p*-Value
Variables			Lower	Upper	
Age (<50 vs. ≥50 years)	0.583	–	–	–	–
Tumor size (T1 + T2 vs. T3)	0.255	–	–	–	–
LN infiltration (yes vs. no)	0.366	–	–	–	–
HER2 (high vs. low)	0.858	–	–	–	–
Histologic grade (G1 + G2 vs. G3)	0.01*	0.17636	0.053442	0.58199	0.00439*
Lymph node status (I + II vs. III + IV)	0.027*	1.233392	0.107277	14.18063	0.866305
TNM stage (I + II vs. III)	0.027*	0.552771	0.042416	7.203702	0.65087
ER (high vs. low)	0.007*	1.589081	0.494627	5.105217	0.436691
PR (high vs. low)	0.009*	2.008442	0.615829	6.550265	0.247599
CXCL1 expression (high vs. low)	< 0.001*	0.396269	0.178428	0.880073	0.02298*

^*^p < 0.05, statistically significant prognostic factor identified by univariate/multivariate analysis

## Discussion

Stromal cells, cytokines and the extracellular matrix contribute to the TME and recurrence or metastases in various types of malignancies. Macrophage regulation is thought to be promising for metastatic monitoring or treatment. Macrophage infiltration creates inflammation that supports cancer initiation and secrets multiple cytokines to promote cancer angiogenesis and invasion and suppresses antitumor immunity^22^. Signaling interactions between macrophages and cancer cells may aggravate the M2 phenotype transformation and induce formation of a pre-metastatic niche^23^. For example, mesenchymal-like breast cancer cells secrete granulocyte–macrophage colony-stimulating factor to promote TAM recruitment, but CCL18 secreted by TAMs may activate the cancer cell EMT program, creating a positive feedback loop between stromal macrophages and cancer cells^14^. Therefore, the identification of key signals in this loop is of interest for future therapies. Well-known secreted proteins, such as CA125, CEA, CA199, AFP and PSA, are biomarkers for monitoring cancer recurrence or metastasis in clinical settings, and proteins in the culture supernatant reportedly reflect the biological behavior of tumor cells in vivo;^24^ thus, the analysis of proteins secreted by stromal macrophages may help identify potential diagnostic and prognostic cancer biomarkers.

Recent evidence has shown that CXC chemokines are keys to malignant initiation and cancer progression, in addition to their role in inflammation. CXC chemokines may be classified based on the presence/absence of a Glu-Leu-Arg ELR motif before the first cysteine amino-acid residue in the primary structure^25^. ELR^+^ CXC chemokines include CXCL1, CXCL2, CXCL3, CXCL5, CXCL6, CXCL7 and CXCL8;^26^ and ELR^-^ CXC chemokines include CXCL4, CXCL9 and CXCL10^27^. The biological functions of CXC chemokines have been described in multiple malignancies. For example, CXCL2 secreted by bladder cancer cells participate in recruiting myeloid-derived suppressor cells and is correlated with a poor prognosis^28^. CXCL3 overexpression is a potential target for breast and prostate cancers^29,30^. CXCL5 silencing may decrease the metastasis and invasiveness of colorectal, liver, bladder, gastric and breast cancers^31–35^. Interestingly, dual roles for ELR^-^ CXC chemokines have been depicted in various cancers. For example, although CXCL4 inhibits angiogenesis, it is protumorigenic in pancreatic ductal adenocarcinoma^36^. CXCL1 is involved in multiple cancer biological processes including angiogenesis, metastasis, tumor growth and chemoresistance^37^. Here, we showed that CXCL facilitated breast cancer lung aggressiveness in vitro and in vivo but had little effect on breast cancer cell proliferation, indicating that CXCL1 can independently enhance breast cancer cell motility. We also identified CXCL1 as the most secreted cytokine from TAMs, and CXCL1 reduction using neutralizing antibody or shRNA in TAMs blocked breast cancer cell metastasis in vitro and in vivo, suggesting a role for TAM-secreted CXCL1 in mediating metastasis and its potential as a therapeutic target. Clinically, CXCL1 has been implicated in breast cancer lymphoid metastasis and poor OS, so it may be a prognostic biomarker of breast cancer.

Previous studies have indicated that multiple mechanisms are involved in CXCL1-induced cancer metastasis. CXCL1, a chemotaxis-stimulating factor, recruits various stromal cells into tumor surroundings to create a pre-metastatic niche to support cancer growth, angiogenesis and metastasis. Such stromal cells include myeloid-derived suppressor cells, bone marrow-derived mesenchymal cells and regulatory cells. CXCL1 can also activate the integrin β1/FAK/AKT or FAK-ERK1/2-RhoA signaling pathway to promote gastric cancer cell migration to the lymphatic system^38,39^. In addition, the metastatic-related genes *EGFR* and *MMP-13* are downstream targets of CXCL1^39^. Furthermore, CSCs are thought to be the driving forces of metastasis, and several CXC chemokines, such as CXCL10, 11 and 12, are regulators of stem cells^40,41^. However, we found no direct correlation between CXCL1 and breast CSCs using CD44^+^/CD24^-^ staining and ALDH assays, indicating that the metastatic-promoting effects of CXCL1 were not attributed to CSC stimulation. Based on the EMT induction effects of CXCL1, a panel of EMT-related genes was selected to identify the novel mechanisms underlying CXCL1-mediated metastasis. SOX4 was validated as the most responsive gene as SOX4 expression was significantly elevated in numerous malignancies, such as colorectal, liver and breast cancers^42^. The high expression of SOX4 usually induces the EMT and indicates a poor prognosis in breast cancer^43^. We found that SOX4 silencing blocked the metastatic indcuing effects CXCL1, indicating that SOX4 contributes to CXCL1-mediated bioactivity. However, many aspects of SOX4 regulation are poorly understood. Here, we showed that NF-κB acts as an upstream regulator of SOX4, and that CXCL1 administration activated p65 phosphorylation and nuclear transportation, which subsequently triggered SOX4 transcription. Inhibition of the NF-κB pathway blocked SOX4 activation and the EMT induced by CXCL1, confirming that CXCL1 induces SOX4-mediated metastasis via NF-κB activation. Our data are in agreement with recent literature. Kuo’s group reported that CXCL1 activates the NF-κB/HDAC1 pathway in prostate cancer, and that NF-κB can act as an upstream regulator of CXCL1^44^. NF-κB-mediated CXCL1 production contributes to maintenance of bone cancer^45^, indicating the existence of a feedback regulation loop between NF-κB and CXCL1.

The results of this study showed that CXCL1 was the most significant cytokine derived from TAMs for inducing breast cancer metastasis, suggesting that the NF-κB/SOX4 pathway may be involved in downstream signaling. Therefore, CXCL1 may be a biomarker for cancer prognosis and a therapeutic target for this disease.

## Materials and methods

### Chemicals and reagents

Recombinant murine or homo CXCL1, IL-4 and IL-13 cytokines were bought from PeproTech (Rocky Hill, NJ, USA). NF-κB pathway inhibitor Bay 11–7082 and macrophage differentiation stimulator phorbol-12-myristate-13-acetate (PMA) were purchased from Medchem Express (Monmouth Junction, NJ, USA). Collagenase IV, Dnase I, gelatin and BCA protein assay kit were provided by Sigma Company (Sigma-Aldrich, St Louis, MO). Bovine serum albumin (BSA) and Coomassie Blue R-250 were bought from Bio-Rad Laboratories (Mexico City, Mexico). ECL Advance imaging reagent was supported by Tanon Company (Tanon Science & Technology, Shanghai, China). The luciferase substrate d-luciferin was bought from Promega (Promega, Madison, WI, USA) and dissolved in phosphate-buffered saline (PBS) at 30 mg/ml for stock at −20 °C.

### Cell culture

Mouse macrophage Raw264.7 and human macrophage THP1 cell lines were obtained from the American Type Culture Collection, and they were cultured in RPMI-1640 medium supplemented with 10% fetal bovine serum (FBS) at 37 °C in a humidified incubator with 5% CO_2_. Raw264.7 cells were induced to M2 phenotype by adding IL-4 and IL-13 co-treatments (10 ng/ml) for 24 h. THP1 cells were induced to attachment by 100 ng/ml PMA and subsequently treated with IL-4 (10 ng/ml) for M2 differentiation. The successful induction of M2 phenotype was identified by flow cytometry analysis. CM was collected as cell culture supernatants in serum-free 1640 medium 24 h after TAMs induction. The mouse breast cancer cell line 4T1, human breast cancer cell lines MDA-MB-231 and MCF-7 were obtained from the American Type Culture Collection. The cells were cultivated in medium (L-15 for MDA-MB-231; 1640 for MCF-7 and 4T1) supplemented with 10% FBS and 1% penicillin and streptomycin (Gibco Life Technologies, Lofer, Austria) at 37 °C in a humidified incubator with or without 5% CO_2_.

### Isolation and in vitro induction of TAMs

The fresh tumor tissues were immediately removed from MMTV-PyVT^+/-^ mice and rinsed in RPMI-1640 medium. The tissues were then cut into small pieces for 1–2 mm^3^ and placed in 5 ml digestive RPMI-1640 (containing 2% FBS, 0.05% collagenase IV, 0.005% Dnase I) with rotating for 2 h at 37 °C, 150 rpm. The cell suspension was then filtered with 100 mesh screen and centrifuged at 50 *g* for 1 min to remove the residual tissue. The supernatants was then centrifuged at 400 *g* for 10 min and the precipitate was re-suspended in RPMI-1640 supplemented with 1% calf serum. The obtained cells were cultured for 1 h at 37 °C with 5% CO_2_, allowing macrophages to attach on culture plates. Unattached cells were removed and the adherent cells were considered as TAMs and would be identified by flow cytometry. This method yielded a relatively pure population of macrophages^46^. Cultured TAMs less than five passages were used for our experiments.

### Flow cytometry analysis

Flow cytometry was applied to analyze the surface markers of macrophages to identify the phenotype of macrophages. Single-cell suspensions were washed in PBS with 2% FBS and adjusted the concentration to 1–5 × 10^6^ cells/ml. For purity analysis of macrophages, cells were incubated with PE-conjugated (P-phycoerythrin) antibody against F4/80 (eBioscience, CA, USA). For M2 surface maker analysis, cells were further analyzed with FITC-conjugated (fluorescein isothiocyanate) antibody against CD206 (eBioscience, CA, USA). All antibodies were used at 5 μg/ml, and the cells were incubated with the antibodies for 30 min at 4 °C and washed with PBS. The samples were fixed with 1% paraformaldehyde and analyzed by using Cytomic FC500 flow cytometry (Beckman Coulter, Inc.). For ALDEFLUOR assay, the experiment was performed using aldehyde dehydrogenase-based cell detection kit (Stem Cell Technologies, Grenoble, France) as described previously. Briefly, 2 × 10^5^ cells were suspended in Aldefluor® assay buffer containing ALDH substrate (Bodipy-Aminoacetaldehyde) and incubated for 45 min at 37 °C. As a reference control, the cells were suspended in buffer containing Aldefluor® substrate in the presence of diethylaminobenzaldehyde (DEAB), a specific ALDH1 enzyme inhibitor. The brightly fluorescent ALDH1-expressing cells (ALDH1^high^) were detected by a 488 nm blue laser. With regard to CD44^+^/CD24^-^ stem-like cell analysis, 2 × 10^5^ breast cancer cells were incubated with FITC-conjugated CD44 and PE-conjugated CD24 antibodies (BD Biosciences, San Diego, CA, USA) for 30 min at 4 °C. After triplicate washes with PBS, the cells were fixed with 1% paraformaldehyde and analyzed by using Cytomic FC500 flow cytometry. A triplicate independent experiment was performed.

### Cytokine array detection

Mouse cytokine antibody array C2 kits were purchased from RayBiotech (Norcross, GA). Briefly, TAMs supernatants from TAMs were collected. The antibody array membranes were blocked in 5% BSA for 30 min at room temperature, cell supernatants were then cultured with antibody arrays overnight at 4 °C and washed for three times. Biotinylated antibody cocktail was then incubated with the membranes for 2 h, followed by signal amplification with horseradish peroxidase -streptavidin. Finally, the signals were detected by chemiluminescence method with ECL Advance reagent and quantified using ImageLab software. A triplicate independent experiment was performed.

### Cell proliferation assay and CXCL1 ELISA detection

MCF-7, MDA-MB-231 and 4T1 cells were seeded onto 96-well plates at a density of 3 × 10^3^ cells per well, respectively. After cell attachment, serial concentration gradients of CXCL1 were added to the wells, with six repeats for each concentration. Cell viability was then detected using MTT (MP Biomedicals, Shanghai, China) according to the manufacturer’s instructions after 48 h. To compare the CXCL1 level in the supernatants of Raw 264.7, M2 phenotype Raw 264.7 and 4T1 cells, a mouse CXCL1 Quantikine ELISA kit (USCN Life Science, Wuhan, China) was applied. The concentration of CXCL1 in the unknown samples was then determined by comparing the optical density of the samples to the standard curve. A triplicate independent experiment was performed.

### Wound-healing and transwell migration assay

For wound-healing assay, 2 × 10^5^ cells were seeded on a 24-well plate. When they grew to full confluence, a ‘wound’ was made in the middle of a culture plate with a 10 μl pipette tip for 4T1, MDA-MB-231 or MCF-7. The wound-healing rate was quantified as the distance of wound recovered vs. that of the original wound at 0, 12 and 24 h. With regard to transwell assay, transwell chambers (8 μm, Milipore, Billerica, MA, USA) were used for cell invasion. The bottom chamber was filled with culture medium containing 10% FBS. In all, 1 × 10^5^ breast cancer cells 4T1, MDA-MB-231 or MCF-7 were suspended in serum-free medium and plated in the upper chamber, respectively. The TAM-derived CM or CXCL1-neutralizing antibody (R&D System, Minneapolis, MN) was added to the bottom chamber to evaluate their influence on cancer cell invasion. After incubation for 24 h, the breast cancer cells were removed from the upper chamber by a cotton swab. Cancer cells penetrated and attached to the bottom of the filter were fixed with 4% formaldehyde in PBS, followed by 20 min staining of 0.5% crystal violet and then subjected to imaging under a 20 × objective. Statistical results of invasion cell numbers were obtained from three independent experiments averaged from five image fields.

### Western blotting

The protein lysates were prepared using RIPA buffer, separated by sodium dodecyl sulfate–polyacrylamide gel electrophoresis gel, transferred to polyvinylidene difluoride membranes (Millipore, Billerica, MA, USA), and probed with primary antibodies at 4 °C overnight. The primary antibodies including vimentin (no. A2666), N-cadherin (no. A0443), Arg-1 (no. A1847), CXCL1 (no. A5802), SOX4 (no. ab80261), p-P65 (no. AP0475), P65 (no. A2547), IKKα (no. A2062), IKKβ (no. A2087) were provided by ABclonal Technology (Cambridge, MA, USA). E-caherin (no. 3195s), p-IKBα (no. 2859), IKBα (no. 4814), p-IKKα/β (no. 2697), β-actin (no. 4970S) and GAPDH (no. 5174s) were purchased form Cell Signaling Technology (Beverly, MA, USA). β-Catenin (sc-7199) and iNOS (bs-0162R, BIOSS) were bought from Santa Cruz (Santa Cruz, CA, USA) and BIOSS (Woburn, MA, USA), respectively. CXCR2 (ab14935) was provided by Abcam (Cambridge, MA, USA). After three washes with Tris-buffered saline with 0.05% Tween-20, the membranes were incubated with secondary anti-rabbit or anti-mouse antibodies (Cell Signaling, MA, USA) for 1 h at room temperature. The signals were visualized using the ECL Advance reagent and quantified using ImageLab software. A triplicate independent experiment was performed.

### Gelatin zymography

Supernatants from 4T1 culture system with or without CXCL1 treatment were collected for MMPs activity analysis by sodium dodecyl sulfate–polyacrylamide gel electrophoresis under non-reducing conditions. One milligram per milliliter of gelatin was prepolymerized on a 10% polyacrylamide gel as a substrate. Electrophoresis was carried out at 4 °C. The gel was washed with washing buffer (50 mM Tris–HCl, pH 7.5, 100 mM NaCl and 2.5% Triton X-100), followed by incubation with a buffer (50 mM Tris–HCl, pH 7.5, 150 mM NaCl, 10 mM CaCl_2_, 0.02% NaN_3_ and 1 lM ZnCl_2_) at 37 °C for 16 h and visualized with Coomassie Blue R-250. A triplicate independent experiment was performed.

### Plasmids, siRNA and cell transfection

The pLent-H1-GFP-Puro-based lentiviruses carrying luciferase were purchased from Vigene Biosciences (Jinan, China) and transfected into MDA-MB-231 cells using Opti-MEM medium containing Polybrene (5 μg/ml), respectively. Recombinant CXCL1 and shCXCL1 plasmids were purchased from Vigene Biosciences and stably transfected into THP-1 cells by LipoFiter^TM^ reagent (Hanbio Biotechnology Co., LTD. Shanghai, China). Scrambled plasmids were set as negative control. SOX4 siRNA, CXCR2 siRNA and their scrambled ones were bought from Invitrogen (Carlsbad, CA, USA) and transfected with X-treme GENE siRNA transfection reagent (Roche Diagnostics, IN) into MDA-MB-231 and MCF-7 cells, respectively. The target protein expression was confirmed by western blotting.

### RNA extraction and real-time PCR analysis

Total RNA in cells were extracted using TRIzol reagent (Invitrogen, Carlsbad, CA, USA) and complementary DNA was synthesized by first-strand CDNA synthesis kit (Roche, Mannheim, Germany) according to the manufacturer’s instruction. Real-time PCR analysis was performed using a SYBR Green kit (Roche, Mannheim, Germany) on Roche lightcycler 480 detector. The primers for *β-catenin, COX-2, Slug, Snail, SOX4, SP1, Stat3, BMI-1, CDH1, CDH2, cMYC, IEB1, IEB2, Nanog, Oct4, SIRT1* and *GAPDH* were listed in Supplementary Table 1. Ct value was measured during the exponential amplification phase. The relative expression level (defined as fold change) of target gene was given by 2^-△△Ct^ and normalized to the internal control. A triplicate independent experiment was performed.

### Immunofluorescence analysis

Cells (3 × 10^5^/well) were seeded in 24-well plates containing cover slips. The cover slips were then fixed in 4% paraformaldehyde for 10 min and permeabilized with 0.2% Triton X-100. After blocking in 10% goat serum for 1 h, the cover slips were incubated with primary antibodies SOX4 (no. ab80261, ABclonal) or Vimentin (no. A2666, ABclonal) overnight at 4 °C. After removing the primary antibodies and triplicate washes with PBS, the samples were further incubated with secondary fluorescence-labeled antibodies for 2 h at room temperature. Finally, the samples were incubated with 4,6-diamidino-2-phenylindole for nuclear staining and detected under LSM710 confocal microscope (Zeiss, Jena, Germany).

### Chromatin immunoprecipitation (CHIP) assay

In all, 1 × 10^7^ MDA-MB-231 or MCF-7 cells were collected and administrated with 1% formaldehyde for 15 min at room temperature. In total, 0.125 M glycine was added into the system for 5 min. Cells were subsequently scraped and centrifuged at 1000 *g* for 3 min at 4 °C. CHIP assay was conducted according to the manufacturer’s instruction (Beyotime, Nantong, China) by immune-precipitating the DNA targets with NF-κB antibody. The anti-human IgG was set as negative control. The −817 to −806 promoter region of SOX4 was predicted as the binding site of NF-κB by JASPAR database. The region was amplified from the DNA samples using the primer pair: forward 5′-TTACGGAGCACTACCTAATGTG-3′ and reverse 5′-CCTGTAAATCCTGCATAGCC-3′. The PCR products were then subjected to gel electrophoresis and compared between groups.

### Animal experiments

All in vivo experiments were performed according to our institutions’ guidelines for the use of laboratory animals and were approved by the Institutional Animal Care and ethical committee of Guangdong Provincial Hospital of Chinese Medicine. Six weeks old female NOD/SCID mice were raised in Experimental Animal Center of Guangdong Provincial Hospital of Chinese Medicine under specific pathogen-free conditions. For orthotropic xenograft establishment, IL-4-treated THP1 or THP1/shCXCL1 cells (1 × 10^5^) were co-injected with MDA-MB-231 cancer cells (3 × 10^5^) into the mammary fat pads of NOD/SCID mice at the ratio of 1:3. Tumor volume (V) was calculated every 3 days using formula V = (length) × (width)^2^/2. When the tumors grow to indicated days, animals were euthanized and tumors were removed.

For the tail vein injection experiments, IL-4-treated THP1 or THP1/shCXCL1 cells (1 × 10^5^) were intravenously co-injected with MDA-MB-231 cancer cells (3 × 10^5^) at the ratio of 1:3. d-Luciferin (150 mg/kg) was intraperitoneal injected and mice were imaged with the IVIS imaging system (IVIS-spectrum; Perkin Elmer, Waltham, MA) every week to monitor the formation of lung colonization. After 8 weeks, the mice were euthanized and their lungs were removed and compared between groups.

### Immunohistochemistry analysis

Tumor specimens were fixed in 10% neutral-buffered formalin for 24 h, followed by standard tissue processing and embedding. Paraffin-embedded tumor sample sections were cut at 3 μm and dried overnight at 37 °C. The sections were then deparaffinized in xylene twice for 10 min each and rehydrated using a graded series of ethanol. Endogenous peroxidase was inactivated by incubating the sections in 3% hydrogen peroxide for 30 min at room temperature. Antigen retrieval was performed by heating the slides in sodium-citrate buffer. The slides were then subjected to incubation with primary antibodies including CXCL1 (no. A5802, ABclonal), Vimentin (no. A2666, ABclonal), N-cadherin (no. A0443, ABclonal), ARG-1 (no. A1847, ABclonal), SOX4 (no. ab80261, ABclonal) and p-P65 (no. AP0475, ABclonal). E-cadherin (no. 3195s, Cell Signaling) and β-catenin (sc-7199, Santa Cruz Biotechnology) at 4 °C overnight in a moist chamber. DAB detection system (ZSGB-BIO, Bejing, China) was applied as chromogenic agents according to the manufacturer’s instructions. Finally, sections were counterstained using Mayer’s hematoxylin, dehydrated, cleared and mounted before examination. Digital images of stained sections were captured using the BX53 upright metallurgical microscope (Olympus, Center Valley, PA, USA). The commercialized human breast cancer tissue microarray (HBre-Duc140Sur-01, Outdo Biotech, Shanghai, China) was used to analyze the correlation between CXCL1 expression and the clinic pathologic parameters of breast cancer patients. The correlation between CXCL1 and survival benefits were analyzed by Graphpad Prism 6.0 software.

### Bioinformatic analysis

The expression levels of CXCL1 transcript in breast cancer were determined from the Oncomine database (www.oncomine.org). The threshold was set at a twofold difference in expression between cancer and normal tissues with a *p-*value < 0.01. The Sorlie cohort study was extracted from Oncomine for survival analysis. The Pawitan study in Oncomine was selected for the correlation analysis between CXCL1 and BRCA1 mutation status. The Bild study in Oncomine was ectracted for the correlation analysis between CXCL1 and triple-negative status. The Cancer Genome Atlas (TCGA) datasets were analyzed and the figures were generated using the cBio Cancer Genomics Portal (http://cbioportal.org). All TCGA data included in this manuscript are in compliance with the TCGA publication guidelines.

### Statistical analysis

All statistical analyses were performed using SPSS 17.0 software (Abbott Laboratories, Chicago, IL). Student’s *t*-test, one-way analysis of variance or χ^2^ test were performed for comparison among groups. Overall survival time was calculated from the time of pathological diagnosis. Survival curves were calculated using the log-rank tests. Univariate and multivariate analyses (Cox proportional hazards regression mode) were performed to identify the independent factors relevant to patient survival. *p* < 0.05 was considered to be statistically significant.

## Electronic supplementary material

supplementary figure 1

supplementary figure 2

supplementary figure 3

supplementary figure 4

supplementary figure 5

supplementary figure 6

supplementary figure 7

supplementary figure legends

supplementary table 1
