# Supplementary figures and images for: CXCL1 derived from tumor-associated macrophages promotes breast cancer metastasis via activating NF-κB/SOX4 signaling
Source: Cell Death Dis. 2018 Aug 29;9(9):880. doi: 10.1038/s41419-018-0876-3 (PMC6115425; doi:10.1038/s41419-018-0876-3)

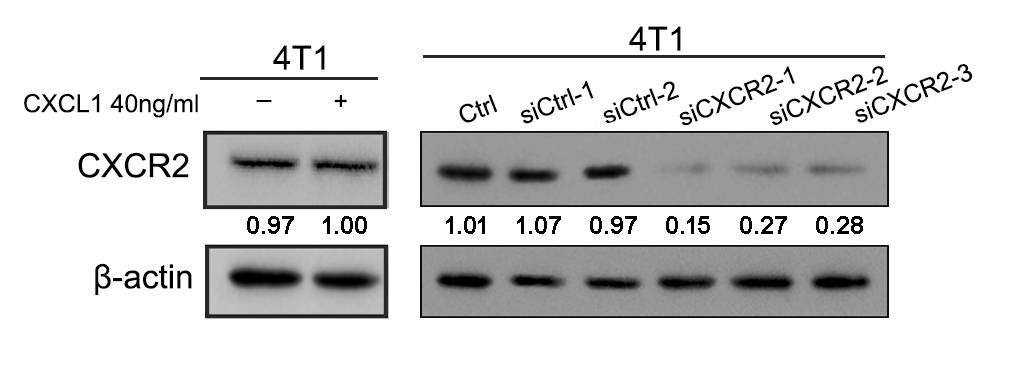

Supplement: Supplementary file 1 — supplementary figure 1 [file 41419_2018_876_MOESM1_ESM.tif]

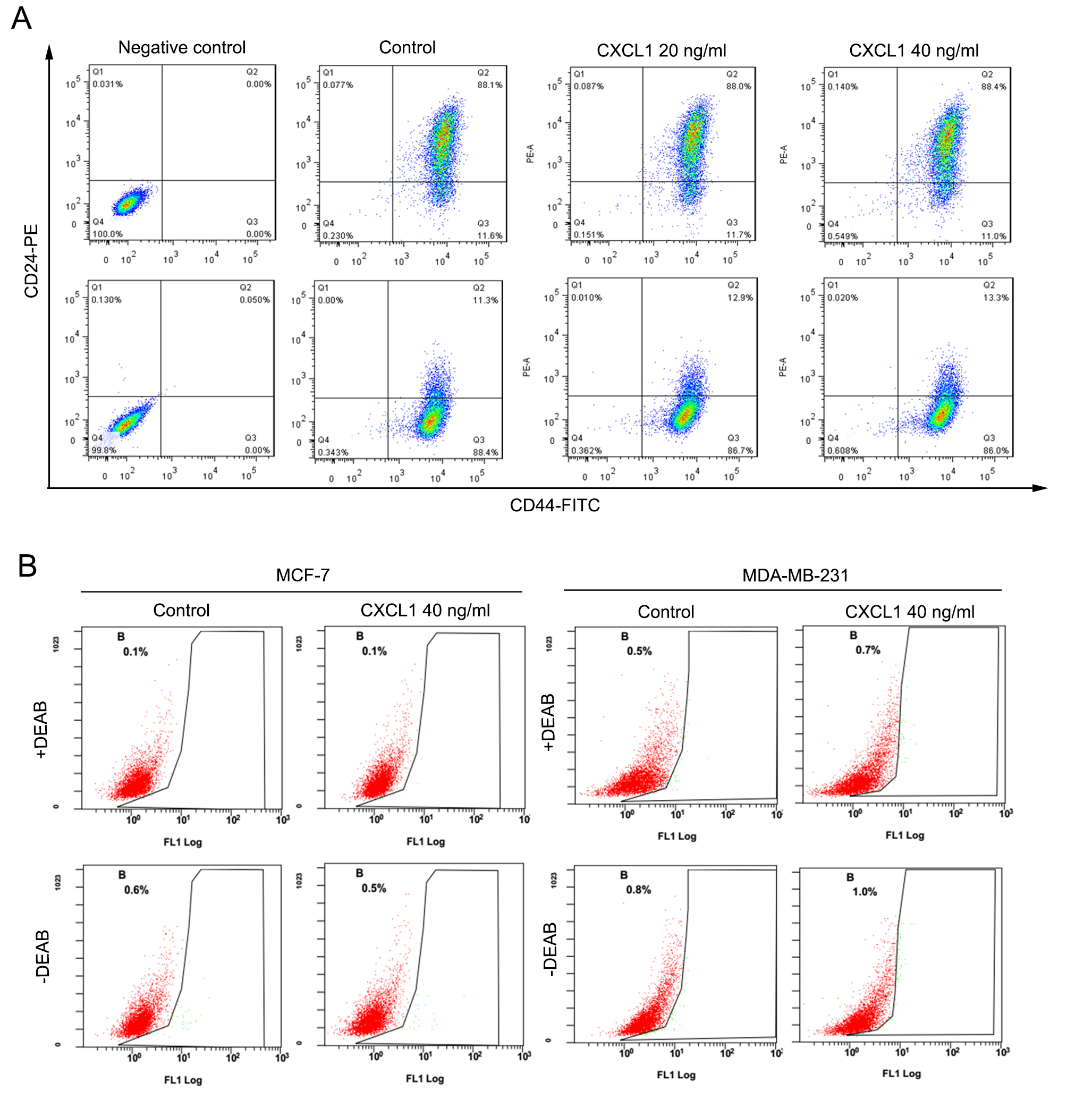

Supplement: Supplementary file 2 — supplementary figure 2 [file 41419_2018_876_MOESM2_ESM.tif]

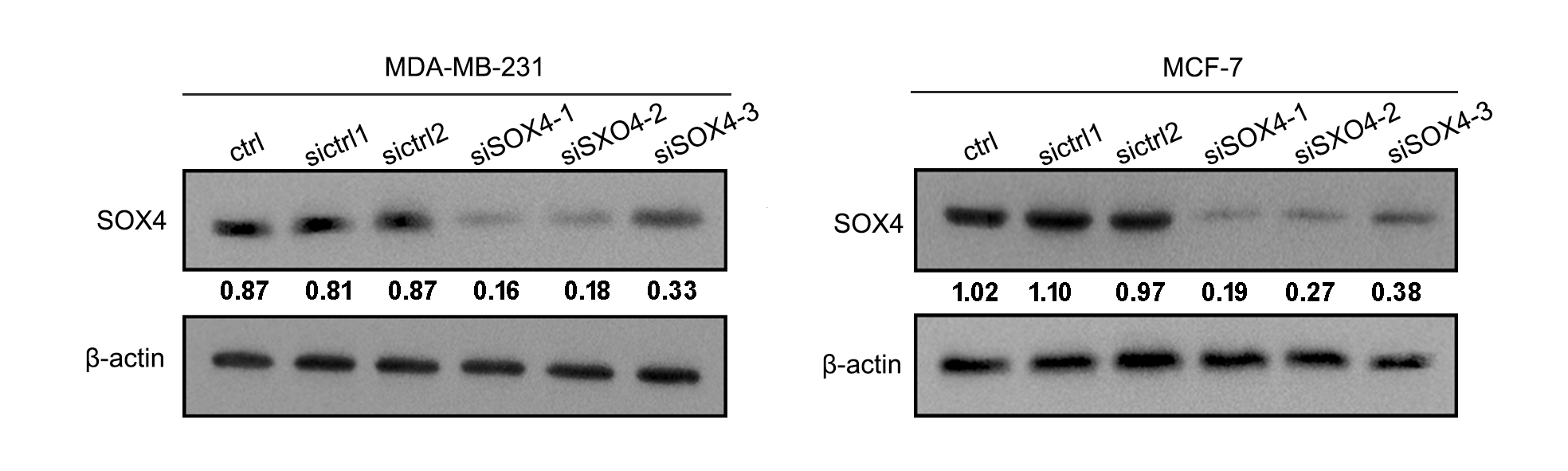

Supplement: Supplementary file 3 — supplementary figure 3 [file 41419_2018_876_MOESM3_ESM.tif]

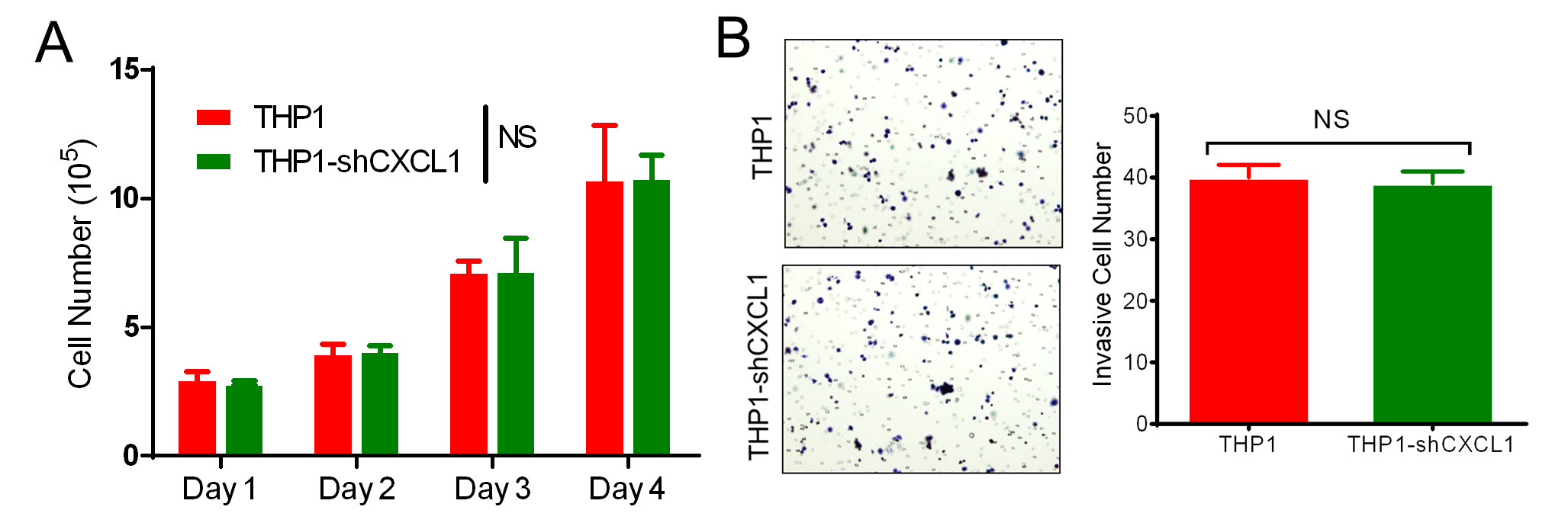

Supplement: Supplementary file 4 — supplementary figure 4 [file 41419_2018_876_MOESM4_ESM.tif]

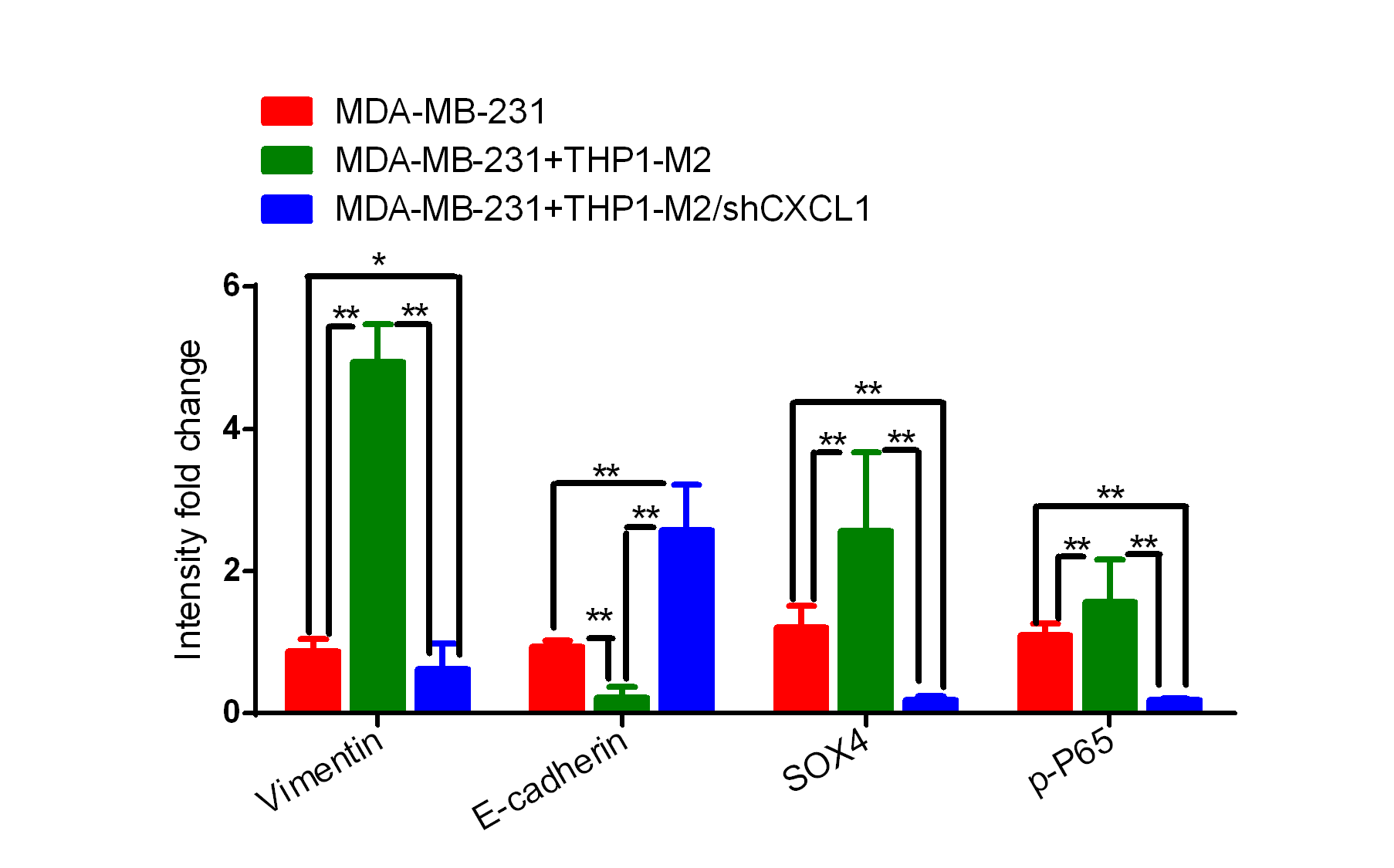

Supplement: Supplementary file 5 — supplementary figure 5 [file 41419_2018_876_MOESM5_ESM.tif]

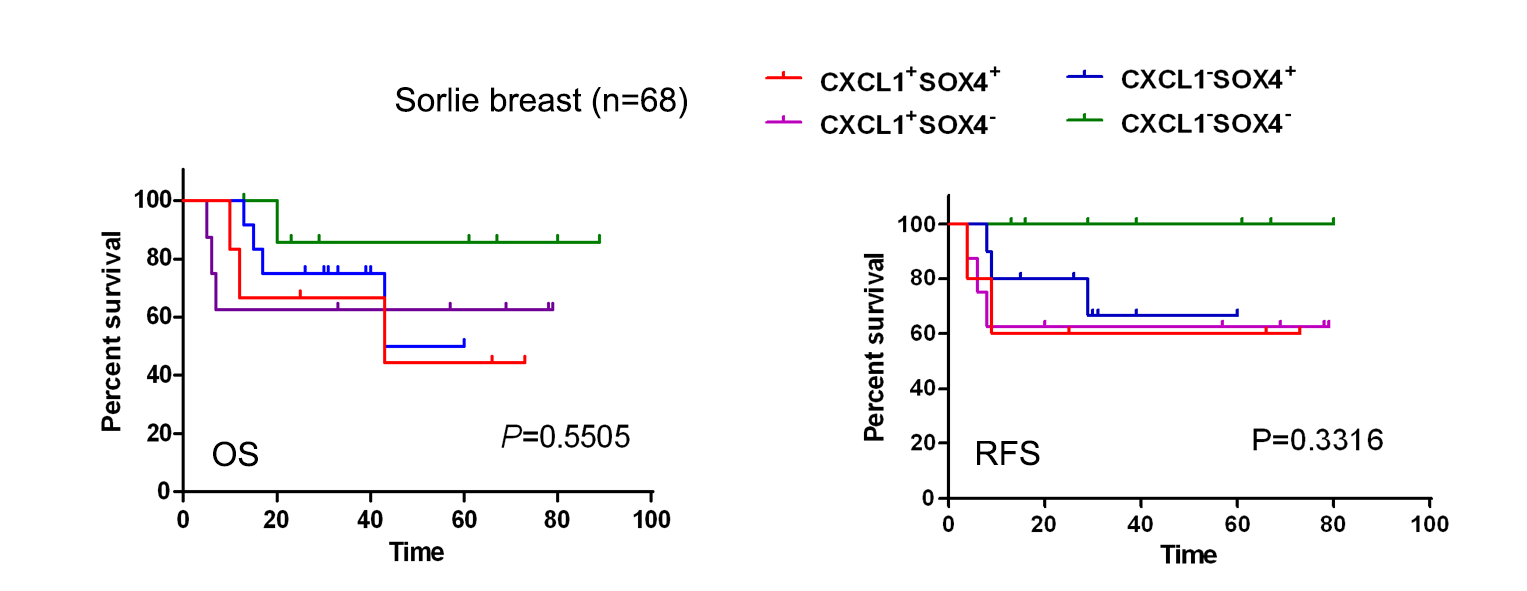

Supplement: Supplementary file 6 — supplementary figure 6 [file 41419_2018_876_MOESM6_ESM.tif]

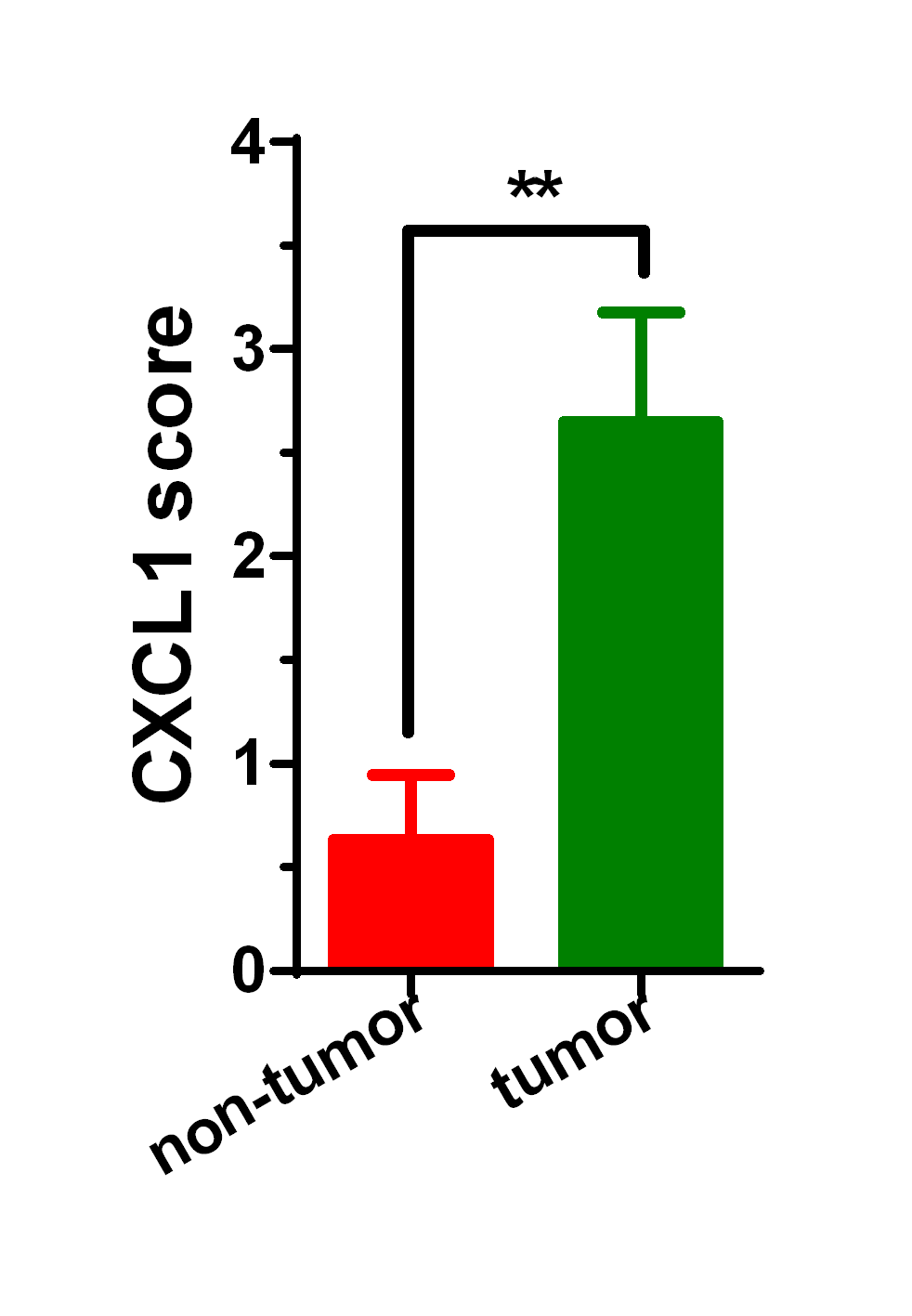

Supplement: Supplementary file 7 — supplementary figure 7 [file 41419_2018_876_MOESM7_ESM.tif]
