# Supplementary material for: CXCL1 derived from tumor-associated macrophages promotes breast cancer metastasis via activating NF-κB/SOX4 signaling
Source: Cell Death Dis. 2018 Aug 29;9(9):880. doi: 10.1038/s41419-018-0876-3 (PMC6115425; doi:10.1038/s41419-018-0876-3)
Supplement: Supplementary file 8 — supplementary figure legends [file 41419_2018_876_MOESM8_ESM.docx]

Supplementary Figure 1 CXCL1 administration does not activate CXCR2 expression in 4T1 cancer cells, and CXCR2 knockdown efficiency using siRNA was validated by western blotting.

Supplementary Figure 2 CXCL1 administration does not increase the population of breast CSCs. (A) CD44-FITC/CD24-PE staining demonstrated that CXCL1 treatment did not induce breast CSCs enrichment; (B) ALDH analysis also revealed that CXCL1 treatment did not increase breast CSCs.

Supplementary Figure 3 SOX4 expression in both MDA-MB-231 and MCF-7 cells were knockdown by using siRNA.

Supplementary Figure 4 CXCL1 silencing in THP1 does not inhibit its proliferation and invasion ability.

Supplementary Figure 5 Statistical differences of Vimentin, E-cadherin, SOX4 and p-p65 between mice groups. (All values from three independent experiments are quantified as Mean ± SD, * *P*<0.05, ***P* <0.01)

Supplementary Figure 6 The combination of CXCL1 and SOX4 positive expression does not bring statistical significance in predicting OS and RFS in Sorlie breast study.

Supplementary Figure 7 CXCL1 expression score is significantly elevated in human breast cancer tissues compared to non-tumor tissues. (All values from three independent experiments are quantified as Mean ± SD, ***P* <0.01)
