# Supplementary material for: CXCL1 derived from tumor-associated macrophages promotes breast cancer metastasis via activating NF-κB/SOX4 signaling
Source: Cell Death Dis. 2018 Aug 29;9(9):880. doi: 10.1038/s41419-018-0876-3 (PMC6115425; doi:10.1038/s41419-018-0876-3)
Supplement: Supplementary file 9 — supplementary table 1 [file 41419_2018_876_MOESM9_ESM.docx]

| Gene list | Primer sequence |  |
| --- | --- | --- |
| *β-catenin* | F:gctttcagttgagctgacca | R:caagtccaagatcagcagtctc |
| *COX-2* | F:cttcacgcatcagtttttcaag | R:tcaccgtaaatatgatttaagtccac |
| *Slug* | F: ccatgcctgtcataccacaa | R: acagtgatggggctgtatgc |
| *Snail* | F:aggatctccaggctcgaaag | R:tcggatgtgcatcttgagg |
| *SOX4* | F: agccggaggaggagatgt | R:ttctcgggtcatttcctagc |
| *SP1* | F:tttggcctcaaaacagaagc | R:ccaaacttcctgtggaagaga |
| *Stat3* | F:ctctgccggagaaacagg | R:ctgtcactgtagagctgatggag |
| *BMI-1* | F:ccattgaattctttgaccagaa | R:ctgctgggcatcgtaagtatc |
| *CDH1* | F:ggtctgtcatggaaggtgct | R :gatggcggcattgtaggt |
| *CDH2* | F:acgctctccctccctgtt | R:ggactcgcaccaggagtaat |
| *cMYC* | F:gctgcttagacgctggattt | R:taacgttgaggggcatcg |
| *ZEB1* | F: cgaaacgcgaggttttgta | R:ctagacaggaaatcccacacaa |
| *ZEB2* | F:ttgctccaagatgtgtgagg | R:tgtggggctccagatatacac |
| *Nanog* | F:atgcctcacacggagactgt | R:cagggctgtcctgaataagc |
| *Oct4* | F:caatttgccaagctcctga | R:agatggtcgtttggctgaat |
| *SIRT1* | F:gccagtggattcgctcttt | R:aatttcatcaccgaacagaagg |
| *GAPDH* | F:gactaaccctgcgctcctg | R:gcccaatacgaccaaatcag |

**Supplementary Table 1:** Primer sequences of gene profile for qPCR screening
